# Fabrication Approaches to Interconnect Based Devices for Stretchable Electronics: A Review

**DOI:** 10.3390/ma11030375

**Published:** 2018-03-03

**Authors:** Steven Nagels, Wim Deferme

**Affiliations:** 1 Institute for Materials Research, Hasselt University, Wetenschapspark 1, B-3590 Diepenbeek, Belgium; steven.nagels@uhasselt.be; 2 IMEC VZW—Division IMOMEC, Wetenschapspark 1, B-3590 Diepenbeek, Belgium

**Keywords:** stretchable electronics, soft electronics, soft robotics, liquid metal, horseshoe, conductive composite, standard test

## Abstract

Stretchable electronics promise to naturalize the way that we are surrounded by and interact with our devices. Sensors that can stretch and bend furthermore have become increasingly relevant as the technology behind them matures rapidly from lab-based workflows to industrially applicable production principles. Regardless of the specific materials used, creating stretchable conductors involves either the implementation of strain reliefs through insightful geometric patterning, the dispersion of stiff conductive filler in an elastomeric matrix, or the employment of intrinsically stretchable conductive materials. These basic principles however have spawned a myriad of materials systems wherein future application engineers need to find their way. This paper reports a literature study on the spectrum of different approaches towards stretchable electronics, discusses standardization of characteristic tests together with their reports and estimates matureness for industry. Patterned copper foils that are embedded in elastomeric sheets, which are closest to conventional electronic circuits processing, make up one end of the spectrum. Furthest from industry are the more recent circuits based on intrinsically stretchable liquid metals. These show extremely promising results, however, as a technology, liquid metal is not mature enough to be adapted. Printing makes up the transition between both ends, and is also well established on an industrial level, but traditionally not linked to creating electronics. Even though a certain level of maturity was found amongst the approaches that are reviewed herein, industrial adaptation for consumer electronics remains unpredictable without a designated break-through commercial application.

## 1. Introduction

### 1.1. Concept of Stretchable Electronics

With the advent of stretchable, and, by extension, soft [1] electronics, the well-known advantages of traditional electronic circuits are unlocked to completely new application areas. By the form factor of soft electronics, conformal electronics also come into play. These can deform along with soft interfaces, such as textiles, skin, even tissue, and, additionally, also with moving parts in devices and robots. Their ability to withstand mechanical load implies higher robustness when compared to the traditional printed circuit boards. Best known examples of new applications lie with soft robotics [2] and human computer interfaces [3,4]. These areas benefit directly from making electrical interconnects, sensors, and even actuators fully stretchable.

Consumers will experience the impact of stretchable and soft electronics research predominantly at the verge between robots and humans. Here, the term robot is used in its broadest possible sense, thus also denoting personal electronics, machinery, and medical equipment, amongst others. Starting with the least far-fetched results. Devices with soft electronics have inherently greater mechanical degrees of freedom, and therefore provide new opportunities of interaction [5]. “Stretch to open browser” and “twist to start camera” have the potential to be as strongly naturalized concepts as “swipe to unlock”. Also included in the current vision for stretchable and soft personal electronics are biomedical smart patches, as an extension to wrist-worn activity trackers. These monitor all bodily functions [6], and communicate with each other via a so-called Body Area Network. A less common result will be seen with persons wearing prosthesis. Through soft electronics these individuals are provided with better correspondence of their prosthesis to the mechanical properties of their residual limb. Additionally, the prosthesis can be equipped with closely integrated, soft sensors [7] for a perception of touch, pressure, and temperature more similar to that of the replaced body part. By extension, this principle can also be applied to robots. Once equipped with a high-resolution artificial skin [8], the clumsy robots, as we know them today, will be capable of far more complex interactions. A huge potential is brought forward from the industrial power of robots when they no longer need to be confined in secured zones, but instead, thanks to their newly acquired sensitivity, can be deployed without any risk in close contact with their surrounding humans. The superlative concerning this concept is then reserved for a robot with the properties, as discussed above, but which acquires its functionality fully through soft components. Power supply, actuation, sensors, logic, outputs, and all the connections between them must all exist out of soft materials. The first soft robots will do away with the current cold, brute, and often intimidating perception of working robots.

### 1.2. Basic Concepts

A number of methods are used to obtain a conductive, stretchable trace. The bloc of all these methods can be reduced into three major trends in the field of stretchable electronics. In a first case, one obtains elasticity by adjusted patterning of intrinsically stiff conductive materials. A second approach disperses stiff conductive fillers in an elastomeric matrix in order to form a stretchable electrically conductive composite. A third method makes use of intrinsically stretchable conductive materials.

#### 1.2.1. Adjusted Patterning of Intrinsically Stiff Conductive Materials

Adjusted patterning of stiff conductive materials in terms of fabrication is still closest to traditional rigid Printed Circuit Board (PCB), and, for smaller feature sizes, silicon wafer processing. It can therefore rely on a high compatibility with current industry. In addition to this, attachment of components and creation of stretchable-rigid interconnects is more evident given the compatibility with solder bonds. Characteristic for this approach is the usage of a zigzag or horseshoe pattern, which functions as a two-dimensional (2D) spring to absorb mechanic stress [9]. Metallic layers however need to be very thin. The result consists of tracks that are stretchable, but cannot be described as ‘soft’.

#### 1.2.2. Dispersion of Stiff Conductive Fillers in an Elastomeric Matrix

A fundamentally different approach lies with the dispersion of conductive fillers (particles, plates, wires) in an elastomeric matrix. With a satisfactory high concentration, being the percolation threshold, this will result in a conductive path from particle to particle throughout the entire composite [10]. Fillers with high aspect ratio are preferred because of their practical advantage in the creation of cross-linked structures. An important aspect of this technique is found in the fact that in addition to the electrical, also the mechanical properties of the composite are strongly influenced with the addition of more filler. Typically, these composites have a relatively high resistance. By their rubbery behavior, however, they can be classified as ‘soft’. 

#### 1.2.3. Intrinsically Stretchable Conductive Materials

A third, emerging category is composed of conductive polymers and liquid metal as intrinsically stretchable conductors. The most popular among these conductive polymers are Polythiophene (PTh), Polyaniline (PANI), and Polypyrrole (PPy). These are ideal for solution processing because they can be adjusted to any desired wet deposition technique by means of a solvent. Their biggest disadvantage lies primarily in their degradation even at small strains. Liquid metals, on the other hand, are encapsulated by elastomers in the creation of a stretchable conductive trace that will only fail if the encapsulant fails. This category is based on Gallium based liquid metals, such as eutectic Gallium-Indium (EGaIn) and Gallium-Indium-Tin (Galinstan), which in opposition to Mercury are non-toxic and, moreover, exhibit a very low vapour pressure [11]. Key for this technology is the Galliumoxide layer that forms and ensures stabilization of fluid shapes that would otherwise be impossible through the effect of surface tension.

### 1.3. Fabrication Routes for Stretchable Electronics

Research efforts are mostly limited to stretchable interconnects or sensors. For commercial applications, however, it is necessary to further develop stretchable electronics to circuit level and later to consumer device level. A whole new set of considerations then comes into play, such as patterning of stretchable traces, inclusion of traditional, rigid components, mechanical transition from soft interconnects to rigid components, level of integration of sub circuits (input/output, power supply, logic, etc.), matureness of the employed technologies, production process yield, and safety of all of the used materials. The most common approach to include traditional components is by reducing the elasticity of the device locally around the component, to prevent a mechanical mismatch. One way of achieving this is by incorporating non-stretchable inclusions around the component. Another way by grouping components on rigid isles, which are interconnected by stretchable interconnects. A less frequently used route, because of its complexity, is the development of stretchable counterparts for the traditional rigid components, such as is already more widely implemented for flexible/bendable electronics, e.g., flexible organic thin-film transistor (TFT) circuits [12], thinned-down silicon chips [13], flexible printed battery [14], and flexible organic light-emitting diodes (OLEDs) [15,16]. The combination of stretchable components with stretchable interconnects would lead to a mechanically simpler story. Examples of stretchable components are stretchable electroluminescent surfaces [17] and stretchable batteries [18]. 

### 1.4. Methods of Validation

The characteristics of a good stretchable conductor depends on the application. For sensors, a high Gauge Factor (GF) or Temperature Coefficient (TC), and some resistivity is desired. Interconnects on the other hand have to be as low in resistivity and as little influenced by their environment as possible. When comparing the communicated properties and their measured value in different papers, a large spread is readily observed. The most commonly used devices that measure properties of stretchable circuits are self-built. Results obtained through them are consequently in a lot of cases not recorded according to a standard. Features reported in literature range from resistance as a function of strain, cyclic strain testing and high-frequency behavior, up until washability of the obtained circuits. Although no specific standard exists for testing combinations of elastomers with elastic conductive structures, one can, however, fall back on standards for the measurement of mechanical material properties of elastomers, on one hand, and on standards for the measurement of electrical properties of conductive structures, on the other for some guidance.

### 1.5. Contribution of This Review Paper

The contribution of this paper is to give an overview of the state-of-the-art for the different trends in the field of stretchable electronics. A distinction is made into three classes: adjusted patterning of intrinsically stiff conductive materials (metallic serpentines), dispersion of rigid conductive fillers in an elastomeric matrix (conductive filler-elastomer composites), and intrinsically stretchable conductive materials (conductive polymers and gallium-based liquid metals). Exceptional in this communication are the literature study report on standard testing, together with discussion of industrial matureness for each of the three classes.

## 2. Insightful Geometric Patterning

### 2.1. Underlying Principle

A first main category of approaches to create stretchable conductors lies with rendering a known conductive material stretchable. Metal is not stretchable by itself, but instead, obtains flexibility when sufficiently thinned down. This obtained flexibility can then be dedicated to accommodate stretchability by folding a trace back on itself multiple times, optionally through smoothened out curves. Tortuosity is the name of the game. The property of a path in which its level of curvedness causes a short bridged distance in spite of a much longer path length is called its tortuosity τ. It is defined as

(1) $$\tau =\;\frac{L_{e}}{L}$$

Equation (1) Tortuosity of a given path with $L_{e}$, the total path length, and *L* the bridged distance between the start point and end point of the path [19]. Historically, the patterning of thin traces of stiff metal into so-called tortuous circuit traces only followed after the first conductive filler-elastomer composites, which will be discussed later. Because of strong similarities between the fabrication process of this technology and the one from standard Printed Circuit Boards (PCB), however, it is covered anteriorly in this communication.

### 2.2. Introduction of in Plane Tortuosity through Serpentines

Gray et al. conceived the idea of creating in-plane 2D mechanical springs folding a trace back on itself multiple times [20]. Such a tortuous circuit trace is then able to cope with tensile loading by partially unfolding itself. Experiments with waveform patterns uncovered a positive influence on increasing the traces’ elongation at break by (a) a doubling of amplitude as compared to wavelength (b) thinner circuit traces, and (c) shorter wavelength. In the study, the maximum reached strain was in the order of magnitude of 50% for gold wave patterns of half ellipses with amplitude 40 μm, wavelength 80 μm, trace width 5 μm and thickness of 5 μm. Later, Gonzalez et al. introduced a horseshoe pattern that simulations identify as the best structure to accommodate unilateral strain uniformly [9]. They introduced the scaling factor R/W where R is the radius of the horseshoe pattern, and W is the width of the trace. Their observation encompassed tension and strain in the metal as being constant when the factor R/W is held constant. Incidentally, a higher R/W value gives rise to a decrease of the induced strain in the metal. A thin circuit trace or larger bending radius is preferred. A 90 μm circuit traces was divided into five parallel lengths of 10 μm. In doing so, an over sixfold decrease of plastic strain was simulated in the metal in comparison to a solid 90 μm trace. Ultimate elongation was in the order of magnitude of 20% for the solid trace. In case of the parallel traces design this was 100%. An important observation from three-dimensional (3D) simulation is that the spring interconnects tend to deform out-of-plane during in-plane uniaxial tensile loading.

### 2.3. Alternative Serpentine Shapes

**Figure 1:**
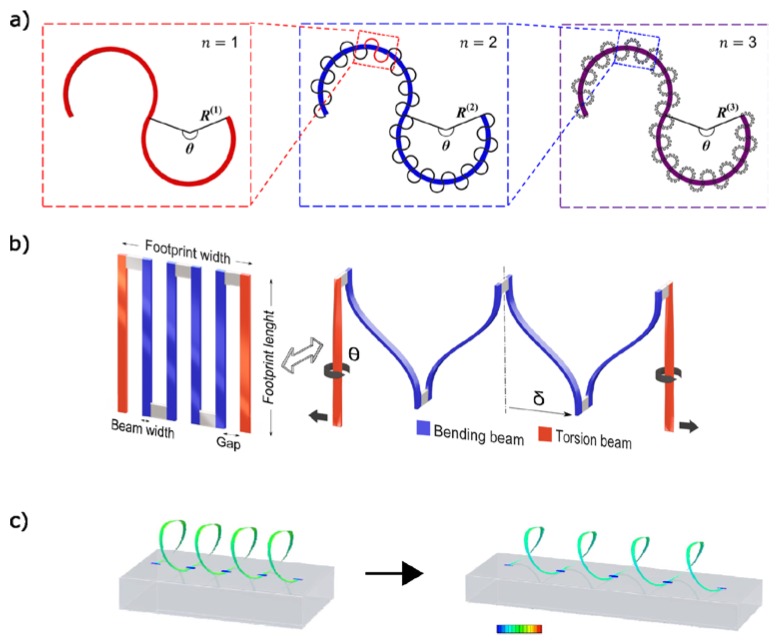
(**a**) typical horseshoe shape along with its higher order fractals (**b**) micro-scale patterned aluminum serpentine (**c**) helices showing gradual increase of built-in tension with mechanical loading. Adapted with permission from [21,23,25].

Horseshoes are typically encountered most in literature. However, promising research meanwhile is performed on alternative serpentine structures that can cope with higher strains. Ma et al. for instance use an approach of higher order fractal patterns. This pattern is visualized in Figure 1a. The unfolding of a serpentine structure is coupled to ever increasing tension [21]. A clear distinction can be observed in stress-strain curves of first, second and third order fractal horseshoe structures when they unfold from low to high order. A scenario of critical strain in the metallic layer was simulated and found to correspond to ca 140%, 400%, and 1200% strain at sample level, respectively, for first, second, and third order fractal horseshoe patterns with θ = 240°, width-to-bend radius ratio of 0.2 and 16 n − 1th order unit cells for each nth order unit cell. Sepulveda et al. abandoned fractal patterns, in favor of a biomimetic approach [22]. A combination of spherulite and lamellar patterns, derived from polymer crystallization, leads to a very intricate design. Silicon nanoribbons of 2 μm width and 30 μm thickness following this pattern were stretched to 490% without failure. The biggest strain reported for a metallic serpentine in the elastic area is, to the extent of the author’s knowledge, at 2040% [23]. The exceptional behavior of the simpler serpentine was explained by the ‘smaller is stronger’ principle for sub-micron features in metal. Figure 1b shows the corresponding pattern. The effective yield strength of patterned aluminum, width 2 μm, and thickness 0.3 μm, was estimated at 700 MPa, about 10× higher than bulk value. A cyclic tensile test of 10 million cycles to 1000% corresponded to a resistance change of about 1%. *L*_0_ of the complete aluminum structure was 17 μm. Kirigami patterns for biaxial strain accommodation were reported by Vachicouras et al. [24]. All of the respective research here reported concerning alternative shapes revolved around structures that were suspended freely in their surroundings. No encapsulation was involved.

### 2.4. Substrate Influence

Due to the high elasticity, one could be inclined towards describing the influence of the substrate as negligible. Xu et al. however did not encapsulate the circuit directly with elastomer, but applied local anchoring to its components in an Ecoflex sub- and superstrate, with the interconnects freely suspended in-between, within liquid Sylgard 184 monomer [26]. The thus obtained circuit had a biaxial ultimate elongation of about 100%, elastic behavior up to 46%, and exhibited a full mechanical decoupling between interconnects and solid-state components. Furthermore, metallic serpentines also do benefit from lamination to a rigid, flexible material such as polyimide (PI) with Young’s modulus (E) 6.18 GPa [27]. Lamination of copper with PI resulted in a delay of crack propagation under cyclic tensile loading. A performance increase of at least a factor of 2 was observed after addition of the PI support layer because it buffers the tension, which is transmitted from the encapsulant onto the copper traces (E = 120 MPa). The encapsulant is a determining factor as a transmitter of tension and possible oppressor of out-of-plane distortion. Pan et al. investigated the role of the encapsulant in tensile loading 100 nm thick copper serpentines sandwiched between 1.4 μm top and bottom supportive PI layers [28]. They found a strong inversely proportional relationship between substrate thickness and elasticity below a certain threshold of thickness. A comparison between Ecoflex 00–30 (E = 60 kPa) and Sylgard 184 (E = 1 MPa) also indicates an increase of the encapsulated serpentines’ elasticity with lower Young’s modulus of the encapsulant. The best result was obtained with 7 μm Ecoflex substrate: 25,000 cycles under 120% strain without any visible micro cracks. Su et al. obtained similar results with thick bar serpentines, which exhibit scissor-like deformation. They extended a serpentine of 45 μm width and 45 μm thickness for 20,000 cycles to 110% without failure [29].

### 2.5. Out of Plane Tortuosity with Buckling

A similar approach makes use of the substrate to accommodate stretch. By applying a pre-tension to this substrate before patterning a conductor on it, the conductor will gain built-in pre-tension after relaxation of the substrate [30,31]. This can be observed from the accordion (buckling) shape which the substrate surface takes on. The wavelength of the obtained corrugated surface is characteristic for each combination of material stiffness of elastomer and metal layer [32]. Tortuosity in this case can be pinpointed to the transversal direction perpendicular to the substrate surface. Before the conductive trace breaks, both the pre-tension in the substrate, as well as the ultimate tensile strength of the metal now has to be overcome. This is similar to the working principle of pre-stressed reinforced concrete. 

### 2.6. 3D Tortuosiy

By combining metallic serpentines with pretensioned substrates, it is possible to achieve 3D structures with high tortuosity. Sun et al. selectively bonded Si (290 nm thick) and GaAs (490 nm thick) nanoribbons (100 μm wide) to a pretensioned Polydimethylsiloxane (PDMS) substrate (4 mm thick) [33]. After relaxation of the PDMS out of plane corrugated nanoribbons where obtained which could withstand up to 100% elongation. The wavelength of the formed 3D serpentines was determined by the prestrain of the substrate together with the distance between the subsequent selective bonding sites. All of the 3D structures were embedded in a covering layer of PDMS. A measurement of resistance change under strain was not reported. The more typical form of a tension spring, the helical form, was obtained in a similar way by Yang et al. [22]. They, however, did not selectively bond straight strips of nanoribbon to the prestrained substrate, but 2D patterned serpentines. These then transformed to free standing helices through relaxation of the PDMS. Finite Element Analysis (FEA) showed that the helices possessed an intrinsic built-in tension over practically their entire trace length, in contrast to the flat 2D ‘seed’ serpentine from which they evolved. The FEA results are depicted in Figure 1c. Under mechanical stress, however, it appears that this built-in tension only increases gradually for the helices. The 2D serpentine, in contrast, has a less preferable distribution of the Von Mises tension. In the corresponding areas of high mechanical stress concentration, increase of this concentration under the influence of strain is of exponential nature.

### 2.7. Transition to Circuits

Production of metallic interconnects is done in most cases via photo lithography at a level similar to that of modern PCB fabrication or integrated circuit manufacturing. Additive approaches utilize electroplating. Tokoro et al. used micro-contact printing to apply necessary seeds prior to the plating step [34]. Kim et al. report findings of similar 2D serpentine designs in the field of printed electronics [35]. When moving to the very small scale, more exotic techniques are used, such as Reactive Ion Etching (RIE) or e-beam evaporation. Going forth to a following level, circuits are made based on these stretchable metallic interconnects. This is predominantly accomplished through utilization of the ‘island approach’. Active components are thereby attached to rigid isles and interconnected with stretchable metallic serpentines [36]. A major advantage of the metallic 2D and 3D spring based traces towards component integration, is their compatibility with soldering processes. Furthermore, they require no custom protective interface to the environment.

### 2.8. Examples of Devices

Various sources report lab-scale stretchable circuits that were based on metallic serpentine interconnects. A flip-chip bonded led array demonstrated the possibilities of lamellar-spherulite stretchable interconnects [24]. Further studies combined rigid solar cells with stretchable interconnects into a stretchable solar cell array [29,37]. Different groups demonstrated the feasibility of bio-monitoring devices, in line with the trend for personal health trackers [22,26,38,39]. A wire-shaped battery was reported based on helical band springs [40]. A final communication reported on foldable transistor arrays [41].

## 3. Conductive Filler Doped Elastomers

### 3.1. Concept of Percolation 

**Figure 2:**
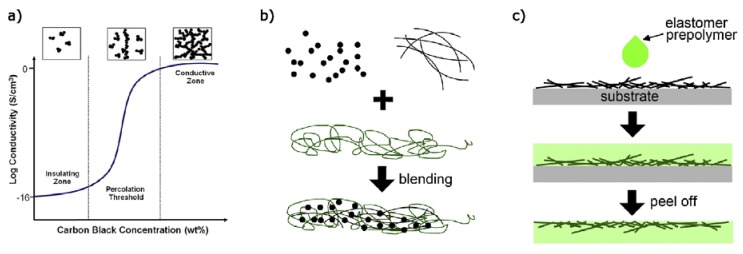
(**a**) typical behavior of conductivity at percolation threshold (**b**) bulk mixing of conductive fillers in elastomer (**c**) surface embedded wires through peel method. Adapted with permission from [42,43].

A second main approach to obtain stretchable conductors, is by rendering elastic materials conductive. As illustrated by Figure 2b, this can be achieved by uniform dispersion of conductive fillers in an elastomer. At sufficiently high volume fraction of conductive fillers, conductive paths will develop from particle to particle throughout the elastomer. The study of development of these paths is the one of percolation theory. Conductivity is gained in a stepwise manner, as depicted by Figure 2a. A clear explanation by Park et al. can be summarized, as follows [42]. The conductivity of the nanocomposite can be determined for any homogeneously dispersed volume fraction of conductive fillers inside when the volume fraction of the percolation threshold is known together with the experimentally determined values for the intrinsic conductivity and the characteristic critical power exponent for a specific type of conductive filler. These last values are strongly dependent on geometry and degree of alignment. There are two mechanisms of conduction. Either by an ordinary metallic conduction via full contacts without contact resistance, either through incomplete contacts by means of tunneling. Specific rules of thumb are stated to lower the percolation threshold based on geometry of the filler. Spherical fillers benefit from small diameters. Plates are as big and thin as possible. Wires are more performant as they exhibit a higher aspect ratio. A precondition however is in effect with the above statements, demanding the complete absence of agglomeration in the suspension [42].

### 3.2. Conductivity-Elasticity Balance

A very important consideration in the making of conductive filler-elastomer nanocomposites is that of conductivity versus elongation [43]. Both of the properties are highly sought after, however an increase of conductivity through an increase of the volume fraction of stiff conductive filler will always result in a reduction of the innate elasticity, and vice versa. Selection of the correct volume fraction therefore is of paramount importance, as confirmed by Niu et al. [44]. An important nuance has to be made by the sometimes much distorted image that a percolation threshold of carbon allotropes, expressed in mass fraction, can create compared to a mass fraction for metallic fillers. This is due to their large difference in mass density. The best comparisons can be made based on volume fraction.

### 3.3. Conductive Fillers

Carbon Black is extracted from the incomplete combustion of organic material. It has a very high surface to bulk ratio, and is traditionally used as a coloring pigment or mechanically reinforcing filler. Specific allotropes also exhibit high conductivity. Their typical particle size is around 100 nm. Percolation threshold lies around 30% by weight—corresponding to about 50% by volume. Resistivities of C-PDMS with the aforementioned particle size range and volume fraction are in the order of magnitude of 10 Ω.cm [44]. 

Carbon nanotubes (CNT’s) are an allotrope of carbon with high aspect ratio. They are characterized by their chirality, diameter, length and number of outer walls. Diameters range from 10 to 100 nm, with lengths of 0.5 to 10 μm. Percolation thresholds vary typically in the order of magnitude of 0.1% to 1% by weight, corresponding to volume fractions of 0.05% to 0.5% [45].

Natural silver is, under normal circumstances, the most conductive material. It is available in flakes varying in order of magnitude from 10 μm to 100 nm. Percolation thresholds vary, depending on particle size, in the order of magnitude of 60% to 80% by weight, in accordance with order of magnitude of 10% to 30% by volume [44]. Resistivities of Ag-PDMS are in the order of magnitude of 0.01 Ω.cm.

Silver nanowires are a more recent geometry of silver with very high aspect ratio. These are typically only embedded on the surface and not mixed throughout the bulk, as is the case for previous fillers. A process is utilized which sequentially consists of depositing a layer of nanowires on a flat and smooth substrate, coating silicone layer on top, which infiltrates the porous nanowire network, and, finally, peeling the elastomer with surface embedded nanowires from the smooth substrate. Figure 2c shows such an infiltrated surface composite. The conductivity of nanowire thin films is usually expressed as sheet resistance in Ω/□, instead of the bulk resistivity Ω.cm. This technique is, to a lesser extent, also applied to CNT’s [46]. Their diameter is in the order of magnitude of 100 nm, while the length can vary between orders of magnitude 10 and 100 μm. Depending on the amount of sprayed Ag NW’s, sheet resistances fluctuate from < 1 Ω/□ to 100 Ω/□ [47].

### 3.4. Ways to Determine Bulk Conductivity of Solid Powders

Conductive fillers should, among other criteria, be selected based on their intrinsic bulk conductivity. Nevertheless, this is not easy to determine. Dispersion in a solvent and subsequent doctor blading will, for instance, result in a layer that varies in thickness and may show significant porosity, rendering it unrepresentative for determining bulk conductivity. Another method is that of powder pressing. Which, in some cases, provides stable pellets from which the bulk conductivity is straightforward to deduce. In the case of carbon black and CNT, however, these pellets are mechanically unstable. Determination of bulk conductivity should therefore be done while the pellet is held under pressure, as illustrated by [48].

### 3.5. Main Ways of Dispersion

Dispersion of conductive fillers in elastomers can be traced back to the 1970s, when carbon fillers were dispersed in silicone rubber [10]. A uniform dispersion is essential to obtain percolation according to the prescribed rules of thumb. The formation of clusters corresponds to dispersing larger fillers, which leads to a higher percolation threshold. Suboptimal dispersion thus nullifies the more advantageous properties of smaller fillers together with their substantially higher required investment. Techniques for successful dispersion of micro- and nanofillers are: solution mixing [49,50], roll milling [51], jet milling [42], high shear force dispensing [52], and planetary gravity mixing [44]. For solution mixing, it is common to both dilute the silicone elastomer and pre-disperse the fillers in solvents such as acetone, hexane, toluene, and xylene. Pre-dispersion of fillers through ultrasonication has a destructive effect on filler geometry. A decrease in diameter and length was recorded for ultrasonic dispersion of CNTs [51]. Metal fillers sometimes undergo a series of washing steps with acetone, ethanol, and DI water [44]. Other conductive filler pre-treatments include UV/O_3_ treatment, ball milling, and silane treatment [53]. 

### 3.6. Patterning Methods

Resulting viscous pastes from the above techniques lend themselves extremely well to patterning in 2D through printing methods such as stencil [54], or screen printing [18,42,55]. A transition to 3D printing can be attained by way of syringe based extrusion printing [49,50,56]. Laser cutting furthermore permits the creations of patterns of thin, cured nanocomposite layers [38]. Niu et al. report minute microstructures that were achieved by combining lithography with molding [44].

The order in which conductive nanocomposites and their possible encapsulant are cured has a considerable effect on the nanocomposites conductivity. Tavakoli et al. carried out a study on the influence of diffusion on the performance of conductive PDMS nanocomposites [49]. Diffusion behaviour must be kept in mind both while choosing the volume fraction of conductive fillers, as well as during patterning and encapsulation steps. First, the volume fraction of conductive filler is best chosen with adequate margin beyond the percolation threshold. This to prevent high sensitivity to the diffusion mechanism of an otherwise fragile percolation network. On a second note, thickness and width of conductive PDMS (cPDMS) traces should be kept high when compared to diffusion depth on one hand. On the other hand, the order of curing between cPDMS and PDMS has to be chosen either so they cure together or so the PDMS encapsulant cures after the cPDMS. Thirdly, curing times should be kept as short as possible relative to the time window within which diffusion can take place.

### 3.7. Achieved Strains

A lot of papers on nanocomposites show no information about their tensile properties. This is because the focus of the study often lies merely with the percolation threshold and dispersion of nanofillers. Valentine et al. reported a conductivity of 0.1 S/cm at 200% strain for a 36 vol % Ag-TPU composite with initial conductivity of 10,000 S/cm [50]. A surface embedded AgNW-PDMS sample of Amjadi et al. exhibited a sheet resistance of 2.2 Ω/□ at 50% elongation [47]. The initial sheet resistance was 1.1 Ω/□. A commercial Ag micro flake based ink was used by Suikkola et al. to screen print structures with 1000 S/cm at 74% elongation and initial conductivity 43,000 S/cm [55]. The Ag microflake-fluorine rubber composite of Matsuhisa et al. achieved a similar 935 S/cm, though at 500% elongation, and with an initial conductivity 4000 S/cm [57]. One of the few bulk nanowire composites, of 20 wt % CuNW with SBS rubber, reached a fracture strain of 920% without a reported change in resistance and an initial conductivity of 217 S/cm [52]. Conclusively, surface embedded CNTPDMS reached 1380% stretch with a 16-fold resistance change without reported initial conductivity [46]. Hwang et al. report an extensive table for further comparison [51].

### 3.8. Device Examples

Practical applications were also demonstrated for the branch of stretchable electronics based on conductive filler-elastomer nanocomposites. Their simple patterning into long, stretchable traces for instance lends itself well to the creation of wide range strain sensors. Many communications report strain sensors, each differentiating on the employed nanocomposite or underlying measuring principle: resistive [46,47,58] or capacitive [38]. A separate mention is appropriate for a completely stretchable measuring device obtained by direct printing of both the strain sensor, as well as the interconnects of the accompanying readout circuit [50]. In addition, a sensor glove demonstrates the direct printability of stretchable electronics on various substrates [57]. Incorporation of a battery, preferably within a matching stretchable form factor, is often needed. This was also understood by the group that made a fully printed, stretchable, and rechargeable battery [18]. Its capacity at first discharge cycle lies at around 3.6 mAh/cm^2^ at a voltage of 1.3 V with a maximum load of 3 mA/cm^2^.The battery degrades both with mechanical strain, as well as with voltage cycling. After 100% elongation is reached, the same technology, under the same discharge current and in its first discharge cycle, exhibits a higher capacity of about 3.8 mAh/cm^2^, however at a voltage of only 1.1 V. Additionally, smart contact lenses were developed in [59]. Materials of choice were AgNW’s and graphene. As a final example, the need for a built-in battery was bypassed by a passive Radio-frequency identification (RFID) demonstrator [55]. This RFID tag is an example of a fully stretchable device hinged on conductive filler-elastomer nanocomposites.

## 4. Intrinsically Stretchable Conductors

### 4.1. Conductive Polymers

**Figure 3:**
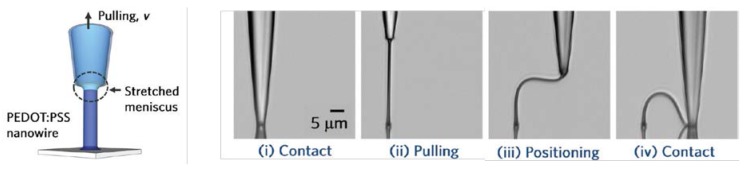
drawing of conductive PEDOT:PSS nanowires. Adapted with permission from [65]. Copyright 2012 American Chemical Society.

Intrinsically, conductive polymers (ICPs) are polymers with conjugated double bonds in the backbone having a conductivity in the range of 10^−9^–10^−6^ S/cm [60]. Polyaniline (PANI), polythiophene (PTh) and polypyrrole (PPy) are the most common known conductive polymers. PANI, PTh, PPy, and their derivatives have an excellent electrochemical activity and good physical and chemical stability [61]. Because of the stiff conjugated aromatic backbone structure, they are not only insoluble and infusible in water and most common organic solvents but their stretchability is also inexisting [60]. This can be overcome by modification of their structure resulting in outstanding optical/electrical properties, good stability, transparency and biocompatibility as shown for Poly(3,4-ethylenedioxythiophene) PEDOT [61]. Its low solution-processability was overcome by doping with poly(styrene sulfonate) (PSS). PEDOT:PPS represents the most successful commercially available ICPs aqueous dispersion today. Upon bending, the resistance is unaffected and no or little cracks are shown. Doping with organic solvents or treatments with particular salts, zwitterions, carboxylic or inorganic acids, polar organic compounds, or cosolvents can further increase the electrical conductivity and the stretchability. Fluorosurfactant dopant, such as Zonyl, can increase the stretching behavior of electrodes [62]. Further research, when combined with the buckling effect of the stretchable substrate led to stretchable organic solar cells [63]. The combination of an intrinsically conductive polymer (polypyrrole) and the buckling method led to highly stretchable electrodes for battery applications [64]. A final approach is the development of highly stretchable fibers based upon these conductive polymers. One research group reported on a direct writing method, as illustrated in Figure 3, to develop such fibers that could be stretched up to 270% without compromising the electrical conductivity [65].

This directly leads to conductive and stretchable yarns that can be applied for textile applications. One approach describes the in situ chemical polymerization of polypyrrole on nylon–spandex stretch fabric to achieve highly stretchable and conductive fabrics [66]. Most of the fibers, however, are a combination of conductive yarns from stretchable materials that are coated with the intrinsically conductive materials [67]. 

### 4.2. Liquid Metal (LM)

Liquid metal (LM) takes over the geometry of its container as it moves in the liquid phase at room temperature. It is intrinsically deformable without deterioration of its mechanical properties, which gives rise to its significance within a stretchable electronics context. The first LM sensors were based on the element Mercury [68], which now is fully depreciated because of its poor wetting and high toxicity. Modern consensus about the term ‘liquid metal’ dictates that it refers to the Gallium-based liquid alloys, namely, eutectic Gallium Indium (eGaIn) and Gallium-Indium-Tin (Galinstan) [69]. This communication will follow that consensus. Circuits that are based on LM must always be fully encapsulated. The metal can then act as a liquid enclosed by a deformable channel. Under deformation, the metal will flow and fully adapt to the new constraints of its container. In this case, the encapsulant will thus usually be the determining factor for maximum elasticity of a LM trace. Elasticity above 700% was reported by Zhu et al. [70]. As the encapsulant here maintains its intrinsic softness (is not made stiff by fillers), and the LM always remains fully compliant, these circuits are deemed to be most appropriately termed as ‘soft’ of all of the preceding. Only with the incorporation of rigid components is the malleability compromised. 

### 4.3. LM Properties

**Figure 4:**
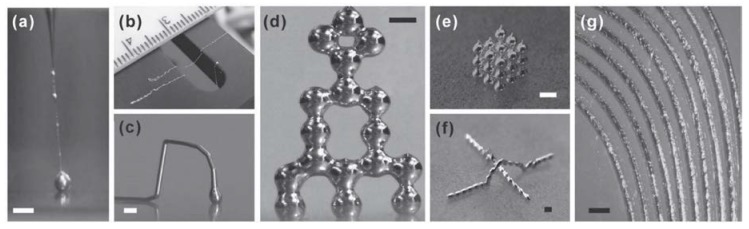
Oxide enabled formation of stable microstructures in liquid metal. Reproduced with permission from [72].

An exceptional property of eGain and Galinstan is their ability to be patterned into stable, free-standing microstructures [11,71]. This is attributed to the presence of a thin layer of Galliumoxide, order of magnitude 10 nm, at the surface. Figure 4 shows thin elongated patterns of LM, which normally would be impossible under the influence of surface tension [72]. 

The material behaves elastic up to a certain critical limit, above which the oxide layer tears, and LM flows without hindrance. The stabilizing contribution of the oxide film is not always desired. If required, it can be disabled by introducing the LM into acidic or alkaline environments. A second special property of liquid gallium alloys is that they display very good wetting towards a wide range of substrates [73]. At the same time, this practically causes difficulties in cleaning out spills. Conductivity is reported as approximately 1/16th of copper [74], which corresponds to about 35–40 × 10^3^ S/cm. In addition LM can be used without health risks. Initial studies even point towards biocompatibility [75]. Reactivity of gallium towards aluminum is high [73]. The gallium-aluminum amalgam has severely weakened mechanical properties compared to bulk aluminum. Potentially this effect also comes into play when aluminum comes into contact with LM as Gallium separates from the alloy.

### 4.4. Patterning of LM

Patterning LM is straightforward owing to both its good, and actively controllable, wetting on a wide range of substrates and its ability to flow [76,77]. Methods that are discussed in this communication are: microchannel filling, stencil printing, laser patterning, selective dewetting, transfer printing, microcontact printing, ink jetting, and emulsion printing.

The creation of very intricate microchannels in PDMS has already been mapped for biomedical applications in the field of microfluidics. When the analytical liquid is replaced by LM [11], one obtains a resistive microstructure, which, among other things, can directly act as a strain sensor or antenna [78]. The advantage here lies with its simple approach. Coarse 2D microchannels are easy to prototype with laser engraving. The biggest obstacle to this is, however, the filling of the microchannels. As was previously stated, an oxide skin resides on the surface of LM. This must first tear under the influence of external force, so the LM may flow. Consequently, as the channel diameter becomes smaller, a higher external pressure must be created for the LM to flow. This is very difficult to practically realize when aiming for thin, flexible microstructures. Connecting a rigid needle or thin, flexible tube to a thin elastic microstructure without high pressure leakage, is very challenging. A solution was recently put forward by means of a vacuum filling method [79]. A second handicap of injecting microchannels with LM to create stretchable electronics is that each individual circuit trace must be filled seperately, and that, to achieve this, an in- and outlet must be provided every time. The injection of LM in very long micro tubes has already been successfully harnessed for textile applications [80]. The introduction of porosity to PDMS, followed by impregnation with LM, is also employed to create an intrinsically conductive material. These LM sponges require further investigation towards appropriate patterning methods and encapsulation [81].

Complementary to first patterning cavities in the elastomer and subsequently filling them with LM, a uniform layer of LM can also be applied to an elastomeric substrate, subtractively patterned in 2D, and afterwards encapsulated to achieve stretchable conductive structures. The uniform layer can be obtained by the use of a roller, or airbrush. Various approaches to subtractively attain 2D patterns are reported. The most simple is by means of stencil printing [54]. A stencil is thus placed on the substrate surface, followed by the application of a uniform layer of LM on top and the removal of the stencil along with the excess of LM. This is a rather manual process which requires a few meticulously performed steps. A more automated approach may come forth from the usage of laser cutters. On one hand, it is possible to encapsulate a uniform layer of LM in PDMS and pattern the entirety of it by using CO_2_ laser cutting [82]. Counterintuitively, even though a CO_2_ laser cannot cut LM directly, the approach succeeds. Key to the underlying mechanism is evaporation of the underlying PDMS accompanied by an increased local pressure that pushes away the LM. Formation of an oxide layer stabilizes the interruption. The result is a cut which effectively runs through the entire PDMS-LM-PDMS stack. An operator removes the excess cut out parts and fills up the voids with pure PDMS. This approach results in very detailed patterns, but still requires manual intervention to remove the undesired pieces. Fully automatic patterning of a uniform layer of LM requires a specific laser for cutting and engraving metal. Patterning LM with a UV laser cutter was demonstrated and was proven to be a fully automatic approach [83]. A different, less direct, approach makes use of the controllable wetting of LM. A circuit pattern is then applied to the substrate surface as microgrooves. These are sprayed with LM until the cavities are filled and the surface is uniformly coated. Finally, the sample is dipped in NaOH. The LM will dewet, but first at the large free surface and only later at the microgrooves. Provided that the correct timing is employed, a selective dewetting thus takes place, according to the applied microgroove pattern [84].

Alternatives to the subtractive patterning methods are found with additive patterning methods. One of the most direct and simple approaches is to load a roller ball pen with LM [85]. A very fine dispersion is thereby established through a well-known and tested principle. Elaborate patterns can be applied directly to the elastomer surface. Automation of this principle can possibly be obtained with the help of a plotter. The resulting traces have a width of ca 200 μm with relatively small deviations. Another manual way of patterning is through transfer printing [86]. Micron scale line widths can be obtained. Coarse automated patterning of LM is achievable via microcontact printing [87]. A specialized tapping mode desktop printer for personal electronics was developed to accomplish complicated patterns [88,89]. Direct jettability of pure LM was furthermore explored in context of inkjet printing [90]. Inconvenience of the oxide layer towards droplet formation was observed. Therefore, an acidic environment is employed to eliminate the oxides’ stabilizing operation, for example, using HCl fumes. Drops of 10 μm size were deposited in a repeatable manner. Minimum feature sizes of 50 μm were thus possible. A more recent approach bypasses the controlled, on-demand separation of LM drops. A micro or nano emulsion of LM droplets in solvent is created by means of sonication [87,91,92]. This emulsion can be rheologically adapted to a specific printing method, which broadens its applicability when compared to the printing of pure LM. After printing the emulsion, the droplets are, however, still separated by their oxide layer. Hence, an additional mechanical sintering step must be administered to rip through the oxide layers and enable the LM droplets to flow into a continuous pattern [93].

### 4.5. Demonstrated Devices

A diversified range of lab scale stretchable circuits based on LM were reported. First and foremost are LM based strain sensors [80,94,95,96]. An extension to this additionally measures, apart from strain, torque, and touch [97]. Another approach functions as a tactile skin for prosthetics [7]. Moreover, temperature sensing was reported [98]. Stretchable antennas can easily be varied in length to change their frequency characteristics [99]. The segment of actuators, then, is represented by an audio speaker [100] and microfluidic pumps [101,102]. In a complementary study, self-propelled LM drops are viewed as engines [103]. A large research effort was provided for miniature scale LM switches [104,105,106,107,108]. In the electronic controlled variants of these LM switches lurks the potential to be used as transistor-like building blocks for soft integrated circuits. Finally, the combination of LM inclusions and elastomer lends itself excellently as a soft heatsink [109].

## 5. Standard Tests

### 5.1. Need

One predominant deficiency throughout communications on stretchable electronics properties seems to rest on standardization. Both in terms of testing as in communication thereof, properties consistently differ in selection or, if selected equally, come forth from a different experimental foundation or with too little background information. All of the reported values are therefore only comparable up to a certain level and should be treated as indications of the order of magnitude for a certain property. Communicated tests in literature are all variations of either resistance vs strain until breakage or cyclic resistance vs strain. One further variation is the pull-restore cycle frequency test [44]. Communicated properties range from initial conductivity and Young’s modulus up to maximum elongation, fracture strain and critical strain, each with its respective conductivity value. Resistance, volume resistivity, and surface resistivity, as well as their conductivity reciprocals are furthermore used alternately without suitable motivation. This makes for a wide variety of communicated properties with no uniform baseline for comparison between them. A suitable standard for electromechanical characterization of stretchable conductors has not come together yet. Both of the constituents, tensile testing, and resistivity measurements, however, are standardized. A uniform baseline thus is already presented and should preferably be attained when reporting on electromechanical behavior of stretchable conductors.

### 5.2. Tensile Tests

Both the American Society for Testing and Materials (ASTM) and the International Organization for Standardization (ISO) have each reported a standard towards tensile properties of vulcanized rubber and thermoplastic elastomers. In ISO 37 [110] and ASTM D412 [111] guidelines are provided on: tested properties, equipment requirements, sample preparation, testing procedure, measurement processing, and contents of the test report. Both offer definitions, formulas, and association to the performed test of: tensile strength, elongation at break, stress at a given elongation, and stress at yield. ISO 37 explicitly illustrates elongation at a given stress and elongation at yield separately. ASTM D412 exclusively handles tensile set—the extension remaining after a specimen has been stretched and allowed to retract. Sample shapes along with their required standardized tooling are described. Within the field of research, products are rarely targeted and custom samples are created in practically any case. Therefore, the majority of stretchable electronics mechanical behavior testing would be served by utilizing so called dumb bell shaped specimen, as opposed to straight or ring specimen. Both of the standards describe well-defined dumb-bell shaped variants. Since geometry is decisive on mechanical dynamics during test, and therefore on outcome, a single variant should be selected for cross communication reference. Other factors to keep constant are: sample thickness and grain orientation, minimum and maximum conditioning times, sample dimensioning methods prior to test, gripper type, specimen insertion depth, rate of elongation, amount of pre-stress, temperature, and humidity. ISO 37 has stricter test report requirements compared to ASTM D412. Median values are reported.

### 5.3. Resistance Measurements

For resistance measurements, only ASTM guidelines were considered. From ASTM D257 [112], a series of apparatus are presented for specific volume resistivity measurements. These can be applied to conductive materials that are utilized in stretchable electronics research to establish their bulk resistivity as stated in ASTM D4496 [113]. In most cases, separate leads for current and voltage are chosen for optimal accuracy. With the addition of ASTM D4496 guidelines, the preferred testing circumstances are further clarified. Since most materials exhibit adequately high resistance temperature coefficients, a single standard measuring temperature is required. Conditioning of samples to this environment is therefore mandatory. Electrification time is kept below 1 min and power input to the test specimen shall never exceed 1W. A minimum of five samples is required, of which average values are reported. ASTM B193 [114] explicitly adds spacing guidelines of voltage relative to current contact points. It furthermore adds the requirement of taking a second reading with current reversed to eliminate errors due to contact potential.

## 6. Discussion

The different techniques to achieve stretchable electronics as introduced above vary in multiple respects. Differentiators, which are explained here are their mechanical properties, their scope and their accessibility for industry.

In terms of mechanical properties, the circuit softness must be kept into mind along with its extensibility. To compare on a macro-scale: a tension spring is stretchable, but certainly cannot be labelled as soft. The softest of all approaches therefore must be the one of the Gallium-based liquid metals. The limiting factor to softness and stretchability then is the intrinsic behavior of the elastomer. The incorporation of LM has almost no influence, seeing as a liquid it does not exhibit resistance against deformation. Most importantly, traces of LM in elastomer show isotropic elasticity. Regardless of direction some ability to stretch is always displayed. This is not the case for most structures of metallic serpentines. The simplest approaches are only able to accommodate uniaxial strain, and can best be compared to the tension spring: elastic and pliable, but not soft. Deployed geometries quickly become very complicated when they need to cope with more realistic, biaxial, tensile loading. An enormous improvement for the macro behaviors of a device based on metallic serpentine interconnects is possible by allowing for the serpentines to move in the bulk of the circuit. This is, for example, made possible by deploying the serpentines as free-standing structures or by encasing the circuit with an elastomeric membrane filled with a viscous, high dielectric fluid. In the transition between metallic serpentines and LM-based circuits, in terms of mechanical behaviour, belong the conductive filler-elastomer nanocomposites. These are also inherently soft in each dimension, but their stiffness is affected by incorporation of conductive fillers, which is in itself directly correlated to the conductivity of the composite. In any case, a higher stiffness will be obtained from the same elastomeric base through the conductive filler-elastomer nanocomposites technique in contrast to the LM approach.

A next differentiator is the one of scope. In terms of minimum feature size, the metallic serpentines are absolute winners, with smallest dimensions in the order of magnitude of a few micrometers. For LM, dimensions are an order of magnitude higher. Decisive for the minimum feature size of conductive filler nanocomposites are diffusion depth and volume fraction of conductive fillers. A higher volume fraction of fillers enables more intricate features, but limits the stretchability. Inversely, with regard to Large Area Electronics (LAE) the etching or metallization process linked to metallic serpentines brings about some limitations. This requires (depending on the feature size) wafer or panel based processing in chemical baths, and thus lack the upscalability of roll-to-roll processes. Liquid metal and conductive filler-elastomer nanocomposites both can be deposited using printing techniques, and are therefore inherently roll-to-roll compatible. 

From the point of view of the industry, it is interesting to know which technique is easiest to achieve on a large scale. For the manufacturer of traditional PCBs, as well as for chip manufacturers, the metallic serpentines are a further development of existing, widely proven techniques that they already have at their disposal. Only a limited number of steps should be added and a lot of expertise is already present for the majority of the process. Behavior of all the materials is known. A recent major venture, based on stretchable electronics with metallic serpentines—as derived from their publication list—is MC10 (www.mc10inc.com, co-founded by prof. J. A. Rogers, Northwestern University, Illinois). The screen printing process by which conductive filler-elastomer nanocomposites possibly can be deposited also benefits from existing industrial maturity on a large scale. This however cannot be stated for the developed conductive filler-elastomer components themselves. Their material properties are still being studied and mapped. With the help of spin-offs, such as Tacterion (www.tacterion.com, Deutsches Zentrum für Luft- und Raumfahrt (DLR)) and Stretchsense (www.stretchsense.com, University of Auckland, New Zealand), pioneering work in terms of the upscaling of the manufacturing processes, in real-life application reliability, and general industrial maturity of conductive filler-elastomer nanocomposites is performed. Finally, for Gallium-based liquid metals, both the patterning methods, as well as the material in itself are still research topics. Publications from the field hint at a huge potential, but towards production of consumer devices this technology is least mature of all. Especially in terms of reliability, a lot of opportunities for further investigation are still available [1]. Exploring these seems indispensable as a foundation for commercial applications, yet, here too, the path is already being cleared by means of a spin-off: Flexosense (www.flexosense.com, National University of Singapore) means to LM technology what Tacterion and Stretchsense mean to the conductive filler-elastomer nanocomposites. The possible contribution of Flexosense can even be considered to be of higher impact, given the technology it commercializes still stands furthest from industry. A separate mention should be added for Feeltronix (www.feeltronix.com, Ecole polytechnique fédérale de Lausanne, Switzerland), a very recent startup in stretchable electronics. Their business is most likely based on biphasic gallium-gold technology [115], although it was not confirmed by their publicly accessible information. When considering industry, the standardization of characteristic tests for stretchable conductors and devices is of the essence. A set of relevant standards were put forward as a foundation for future guidelines on reporting of stretchable electronics’ performance. A normative test is fundamental to streamline research efforts.

## 7. Conclusions

An overview of the state of the art for the different trends in the field of stretchable electronics was given. A distinction was made into three classes: adjusted patterning of intrinsically stiff conductive materials (metallic serpentines), dispersion of rigid conductive fillers in an elastomeric matrix (conductive filler-elastomer composites), and intrinsically stretchable conductive materials (conductive polymers and gallium-based liquid metals). For each of these classes separately, the current technological characteristics, patterning methods, and recently demonstrated practical applications were discussed. Guidelines from standard testing literature were offered as a foundation for cross community establishment and the communication of comparable figures of merit. The need of a normative test was pointed out, however, left open for community dialogue. A discussion of the technologies in relation to each other was further reported together with an estimate of their industrial maturity. In the future, it is expected that each of these technologies ends up in a specific position in the value chain. A possibility would be that conformability on chip is built in together with a connection fan-out by means of metallic serpentines. Gallium-based liquid metal interconnects should then connect to the contact pads of this fan-out to provide the most compliant mechanical properties over longer distances. The use of conductive filler-elastomer nanocomposites would ultimately be reserved to create conductive interfaces throughout the LM encapsulation, sensors with custom gauge factor, or stretchable batteries.
